# Effect of an intervention to enhance guideline adherence of occupational physicians on return-to-work self-efficacy in workers sick-listed with common mental disorders

**DOI:** 10.1186/s12889-015-2125-3

**Published:** 2015-08-19

**Authors:** Karlijn M. van Beurden, Jac J. L. van der Klink, Evelien P. M. Brouwers, Margot C. W. Joosen, Jolanda J. P. Mathijssen, Berend Terluin, Jaap van Weeghel

**Affiliations:** Tilburg University, Tilburg School of Social and Behavioral Sciences, Tranzo Scientific Center for Care and Welfare, PO Box 90153, 5000 LE Tilburg, The Netherlands; Netherlands School of Public & Occupational Health, PO Box 20022, 3502 LA Utrecht, The Netherlands; Department of General Practice and Elderly Care Medicine, VU University Medical Center Amsterdam, EMGO Institute for Health and Care Research, PO Box 7057, 1007 MB Amsterdam, The Netherlands; Phrenos Centre of Expertise, PO Box 1203, 3500 BE Utrecht, The Netherlands; Parnassia Group, Dijk en Duin Mental Health Center, PO Box 305, 1900 AH Castricum, The Netherlands

## Abstract

**Background:**

Since a higher level of self-efficacy in common mental disorders is associated with earlier return-to-work (RTW), it is important to know if work related self-efficacy can be increased by occupational health care. The primary aim of this study was to evaluate whether an intervention to enhance guideline adherence of occupational physicians lead to an increase in RTW self-efficacy in workers three months later. The secondary aim was to evaluate whether the intervention modified the association between RTW self-efficacy and return-to-work three months later.

**Methods:**

A total of 66 occupational physicians participated in the study. They were randomized into two groups; the intervention group received a training, the control group did not. The training aimed to enhance adherence to a mental health guideline that contained strategies that are supposed to enhance RTW self-efficacy. In 128 sick-listed workers guided by these occupational physicians, RTW self-efficacy, RTW, and personal, health-related and work-related variables were measured at baseline and three months later. Generalized linear mixed models analysis and linear mixed models analysis were used for the evaluations.

**Results:**

In workers whose occupational physicians had received the training RTW self-efficacy increased significantly more than in workers whose occupational physicians had participated in the control group (t = −2.626, *p* ≤ .05). Higher baseline RTW self-efficacy scores were significantly more often associated with full RTW than with no RTW three months later (OR 2.20, 95 % CI 1.18–4.07), but the intervention did not affect this association.

**Conclusions:**

This study showed that a training to enhance guideline adherence of occupational physicians leads to increased RTW self-efficacy in workers sick-listed with common mental disorders during the first months of sickness absence in a real-life occupational health care setting. This insight is helpful for optimizing the recovery and RTW process, and for understanding the role of RTW self-efficacy in this process.

**Trial registration:**

ISRCTN86605310

## Background

Self-efficacy is an interesting factor to consider in the return-to-work (RTW) process of workers, because unlike other factors that predict RTW (e.g. age, gender) it can potentially be influenced. Self-efficacy is the individual’s conviction that one has the ability to successfully perform a certain behavior [1]. According to Bandura’s self-efficacy theory, enhancement of an individual’s sense of self-efficacy is an essential mechanism of change [2]. Psychological interventions arising from this theory are based on the assumption that individuals who are seeking help have a low sense of self-efficacy. The main aim of this help should therefore be to restore self-efficacy. Most effective interventions to restore self-efficacy involve working on one or more of the five elements that construct beliefs that an individual has about his or her abilities: vicarious, imaginal, and performance experiences, verbal persuasion, and physiological and emotional states [2]. Of these five elements, performance experiences have been shown to be the most powerful. For example, success at a task or behavior strengthens self-efficacy expectations for that task or behavior [2].

Recent studies have shown that an individual’s level of work related self-efficacy at the start of the sickness absence is an adequate predictor of time until actual RTW [3–5]. In seeking an understanding of how to facilitate the recovery and RTW process, many studies have tried to identify factors that influence the time until RTW for workers with mental health problems. Frequently identified factors that have been found to be related to later RTW are: depression, anxiety disorders, burnout, co-morbid mental health problems, older age, low education, history of previous sick leave, high job stress, reorganizational stress, threat of unemployment, and part time work [6–15]. Factors related to an earlier RTW include: higher self-efficacy, active problem-solving coping strategies, lower age, frequent communication with supervisor, and quality and continuity of occupational care [6, 8, 3, 16, 17]. With regard to gender, mixed outcomes have been found [6, 11, 12].

The Netherlands Society of Occupational Medicine developed a guideline entitled “The management of mental health problems of workers by occupational physicians” in 2001 and revised it in 2007 [18]. The recommendations and interventions that are included in this practice guideline for occupational physicians address a combination of four of the five elements that are supposed to restore self-efficacy according to the theory of Bandura. In general the use of evidence based guidelines is considered to be effective to improve the patient care [19, 20]. However, previous studies on the use of this Dutch guideline showed that despite their positive attitude towards this guideline, actual guideline adherence of occupational physicians was minimal [21, 22].

For a larger study aiming to enhance occupational physicians’ adherence to this guideline, a training was developed. This training entails techniques for the OP to get grip on the elements and interventions that contribute to enhance work related self-efficacy in workers sick-listed with common mental disorders. Sickness absence due to common mental disorders, such as depression, anxiety disorders, and adjustment disorders, is a problem in many Western countries [23]. Long-term sickness absence in particular leads to substantial individual suffering, and high societal and financial costs [24, 25]. The present study aimed to evaluate the effects of this intervention to enhance guideline adherence of occupational physicians on RTW self-efficacy and on the association between RTW self-efficacy and actual RTW.

### Research questions

1) Does the intervention to enhance guideline adherence of occupational physicians lead to increased RTW self-efficacy in workers three months later, as compared to workers guided by occupational physicians who did not receive the training (i.e. care as usual)?

2) Does the intervention to enhance guideline adherence of occupational physicians modify the association between RTW self-efficacy of workers, as measured shortly after a first consultation with an occupational physician, and actual RTW status three months later?

## Methods

### Design and procedure

This study was part of a larger study on the effectiveness of guideline-based care provided by occupational physicians on the recovery and RTW of workers with common mental disorders. A more elaborate description of the design and procedure of this study has been published elsewhere [26]. In this cluster randomized controlled trial, the participating occupational physicians were recruited from the sites of a large collaborating occupational health service in the southern part of the Netherlands. This occupational health service provided care for a wide variety of companies in wide range of sectors (e.g. health care, education, municipality, engineering, industry). The 66 occupational physicians participated on a voluntary basis and were randomly assigned either to an intervention group (32) or to a control group (34). One occupational physician decided not to participate before the start of the training. The remaining 31 occupational physicians attended all training sessions. After completing the training occupational physicians received educational credits.

Eligible workers were selected from the sick leave registration system of the occupational health service. All workers, aged 18–64, counselled and diagnosed with a mental health problem by to a participating occupational physician were invited to participate after their first consultation with the occupational physician. According to the guideline the first consultation is within two weeks after the first day of sickness absence and it is always within six weeks after the first day of the sickness absence in accordance with the Dutch Gatekeeper Improvement Act [27]. Workers interested in participating in the study were screened by the researchers during a structured telephone questionnaire assessing the inclusion and exclusion criteria (e.g. age, current sickness absence, occupational physician). Workers who met the inclusion criteria were sent a baseline questionnaire shortly after the structured telephone questionnaire. For the purpose of this study, only data on RTW self-efficacy and additional variables relevant for RTW were used. Three months after the baseline questionnaire, workers received a second questionnaire to measure their RTW self-efficacy. Data on sickness absence and RTW were extracted by the collaborating occupational health service from its registration system, or were provided by the human resource management departments of participating companies. Three months after the first consultation of the worker with the occupational physician the status of RTW was measured based on the data of the collaborating occupational health service.

Approval was obtained from the Medical Research Ethics Committee of St. Elisabeth Hospital in Tilburg (MREC number 1162).

### Intervention

#### Training to enhance guideline adherence of occupational physicians

The training was based on findings from the scientific implementation literature on how to enhance guideline adherence [28–30]. Specifically, the focus of the training was on overcoming the three main clusters of barriers to guideline adherence: lack of knowledge, negative attitudes, and practical barriers [31]. During the interactive training, small peer-learning groups of occupational physicians discussed the content of the guideline and to what extent this related to their own practice. During the eight sessions (divided over 12 months), they explored their own barriers for use of this guideline and exchanged ideas to overcome them. A trainer (MJ) guided this training by structuring the peer-group learning sessions, facilitating the discussion, and monitoring the progress. During this training, a Plan-Do-Check-Act approach was used [32]. The OPs learned about the content of the guideline, identified barriers that prevented them from using the guideline, found solutions to overcome these barriers, tried out these solutions in daily occupational practice, and evaluated the tested solutions, and if needed adapted the solutions until they were useful for practice. Occupational physicians in the control group received no training and provided care as usual [26].

The training was provided as planned [Joosen et al., submitted, unpublished observations]. A pre- and posttest difference indicated that the self-reported guideline adherence of the participating occupational physicians (*n* = 31) had significantly improved, and 14 months after the end of the training this improved self-reported guideline adherence stayed constant. Also the number of physicians that reported good adherence to the guideline improved considerably after the training.

#### Content of the guideline

In summary, the guideline includes four consecutive steps [26]. The first step is one of problem orientation and diagnosis. The occupational physician sees the employee shortly after the first day of sick leave (within the first 2 weeks). A simplified classification that classified mental health problems in four categories was introduced: a) stress-related complaints (such as adjustment disorders), b) depression, c) anxiety disorder, and d) other psychiatric disorders. The occupational physician provides a diagnosis. If necessary the occupational physician contacts the general practitioner and refers the worker to a psychologist, psychiatrist or other professional for treatment. Furthermore, the problem inventory focuses both factors related to the worker and the work environment as well as the interaction between these two.

During the second step, called the intervention phase, the occupational physician evaluates the process of recovery in which the problem solving capacity of the sick-listed worker has to be monitored and enhanced. First, the occupational physician provides the worker with information about the recovery process and what is needed for recovery to enhance understanding and acceptance before starting to solve the problems. When recovery stagnates, the occupational physicians uses cognitive behavioural techniques to enhance the problem-solving capacity of the worker and follows the three phase model of the stress inoculation training [33]. The occupational physician uses a variety of techniques based on cognitive behavioral and problem solving therapy that are expected to increase self-efficacy, such as verbal persuasion, imaginal experiences, physiological and emotional experiences and performance experiences. For instance by providing information on mental health problems and their recovery process, stimulating the worker to talk about their problems with others, positively re-labeling the situation, providing assignments that help to structure problems and worries, and providing assignments addressing symptoms, emotions and life style. All these interventions are expected to contribute to the acceptance, recovery, readiness to solve problems, and to a first step in restoring the self-efficacy of the worker. After the crisis phase has become manageable, the occupational physician encourages the worker to invent the factors that obstruct the performance of work tasks, such as factors related to the worker and to the work environment, and to the interaction between both. Finally, the occupational physician encourages the worker to find solutions to solve these problems and to practice these solutions during the recovery process [18]. For example by providing the assignment to make a list of stressors described in concrete (problem) situations and to prioritize in order to solve them, and providing the assignment to make two RTW plans (one easily achievable and one more ambitious) and think through the prerequisites needed to achieve these plans. These assignments are expected to contribute to a sense of increased manageability, an increased problem solving capacity, positive experiences and to a sense of increased self-efficacy.

The third step of the guideline focuses on relapse prevention: integration of relapse prevention from the first contact with the worker by enhancing the problem-solving capacity of the worker. According to the fourth step, a process based evaluation is made: during follow-up meetings evaluation of the recovery process includes the perspectives of the worker, supervisor, and other professionals. Follow-up consultations with the worker takes place every 3 weeks during the first 3 months, and then every 6 weeks thereafter. The occupational physician contacts the supervisor or work environment once a month.

### Measures

RTW self-efficacy was measured at baseline and three months later by the RTW self-efficacy scale for workers with mental problems [4]. The RTW self-efficacy scale is a self-report questionnaire that contains 11 statements about the reporting worker’s job. The worker is asked to imagine that he or she would start working his or her full contract again tomorrow. This is followed by statements such as: “I will be able to perform my tasks at work,” “I will be able to deal with emotionally demanding situations,” and “I will be able to cope with work pressure.” Response categories range from “totally disagree” to “totally agree,” over a six-point scale. The mean score across the 11 items represents the RTW self-efficacy total score. Higher RTW self-efficacy scores indicate higher self-efficacy with regard to RTW. The range of the scale’s Cronbach’s alphas has been shown to be from 0.90 to 0.96 across samples [4], and the RTW self-efficacy scale has been shown to be predictive of an actual RTW within three months [4]. According to the developers of the instrument it can be used over the course of the RTW process, even after workers have fully returned to work [4].

RTW status three months after the first consultation of the worker with the occupational physicians was measured with data extracted from the registration system of the occupational health service. Here, the worker was given the status of full RTW, partial RTW, or no RTW. Full RTW was defined as working the same hours as prior to the sickness absence. Partial RTW was defined as working fewer hours than prior to the sickness absence.

Common mental health symptoms were measured at baseline by the Four-Dimensional Symptoms Questionnaire (4DSQ), a self-report questionnaire that measures the four dimensions of common mental health symptoms: distress, depression, anxiety, and somatization. The 4DSQ consists of 50 items (each scored on a 5-point scale) and refers to symptoms experienced during the past week. The 4DSQ has been shown to be a reliable and valid instrument [34]. The Cronbach’s alphas for each of the four dimensions ranged from 0.84 to 0.90 [34].

Burnout symptoms were measured at baseline by the Utrecht Burnout Scale–General Survey (UBOS), which is the Dutch version of the Maslach Burnout Inventory (MBI). The UBOS is a self-report questionnaire that measures work-related emotional exhaustion, mental distance, and competence. Higher scores on exhaustion and distance and lower scores on competence indicate burnout. The UBOS has been shown to be a reliable and valid instrument [35].

Coping was measured at baseline using the shortened 14-item version of the Utrecht Coping List (UCL), a self-report questionnaire that measures coping style on a 4-point scale. The 14-item version assesses the following dimensions: 1) active problem-focused coping, 2) emotional coping, and 3) looking for distraction and decreasing tension [36].

Psychological job demands, decision latitude, social support (from supervisor and from colleagues), and job insecurity were measured at baseline using the Dutch version of the Job Content Questionnaire (JCQ), a self-report questionnaire that measures the social and psychological characteristics of jobs. The dimensions of the JCQ have been shown to have a moderate to good reliability [37].

The following personal characteristics were assessed during the telephone questionnaire: age, gender, education level (low, middle, or high) [38], and number of working hours per week.

### Data analysis

All analyses were performed with SPSS version 19.0. Descriptive statistics were used to describe the baseline characteristics of the workers.

#### Does the intervention lead to increased RTW self-efficacy in workers?

In order to test for possible differences in RTW self-efficacy at baseline between the two groups, an unpaired *t*-test was performed. A linear mixed models analysis was used to evaluate whether the intervention lead to increased RTW self-efficacy three months later taking into account that having established full RTW could be related to a higher RTW self-efficacy score after three months. The data file was restructured for this analysis, and repeated measures within linear mixed models were used to evaluate the change in RTW self-efficacy between baseline and three months later. The intervention, the measurements and RTW status were added as fixed factors to the model. Moreover, the interaction effects between intervention and measurements and between RTW status, intervention and measurements were added to evaluate whether the change in RTW self-efficacy differs for the control and the intervention group, and whether RTW status influences the change in RTW self-efficacy modified by the intervention. In case RTW status did not have an interaction effect on the change in RTW self-efficacy, the interaction term with RTW status was removed from the model. In case RTW status did not influence RTW self-efficacy, RTW status was removed from the model. The Akaike information criterion was used to test whether it was necessary to control for the multilevel effect of the occupational physicians. The model with the smallest Akaike information criterion represents the best-fitting model, so this model was used [39]. Glass’ delta effect size was calculated to gain insight into the impact of the effect found.

#### Does the intervention modify the association between RTW self-efficacy and RTW status?

Multinomial logistic regression within a generalized linear mixed models analysis was used to evaluate whether the intervention modified the association between RTW self-efficacy at baseline and actual RTW status three months after the first consultation with the occupational physician. In the analysis, “No RTW” was used as the reference category, so both “Full RTW” and “Partial RTW” were compared to the “No RTW” category. The intervention and RTW self-efficacy were added as fixed factors to the model. Moreover, the interaction between intervention and RTW self-efficacy was added to test whether the effect of RTW self-efficacy on RTW status depends on belonging to the intervention group or the control group. The Akaike information criterion was used to test whether it was necessary to control for the multilevel effect of the occupational physicians. The model with the smallest Akaike information criterion represents the best-fitting model, so this model was used [39]. Univariate multinomial logistic regression analysis was used to test for potential confounding factors (age, gender, education level, sick leave history, active problem-focused coping, emotional coping, looking for distraction and decreasing tension, distress, depression, anxiety, somatization, burnout, decision latitude, psychological job demands, social support, and job insecurity) and select those that had an association with the dependent variable RTW (*p* ≤ .05). If an association was found, the potential confounding factor was tested for association with the independent variable RTW self-efficacy (*p* ≤ .05). If the potential confounding factor had an association with both the independent and the dependent variable, it was considered to be a confounding factor for the association between RTW self-efficacy and RTW and was added to the model.

## Results

### Study population

In total, 128 workers participated in this study. Their baseline characteristics are presented in Table 1. Sixty percent of the participating workers were female. About two-thirds of the workers were highly educated. The mean number of contracted working hours was 32 h a week. On average, workers had moderately increased distress complaints. Overall 28.9 % of the workers fully returned to their work and 22.3 % of the workers returned partially three months after the first consultation with the OP. Table 2 presents the RTW self-efficacy means per RTW status in the intervention group and in the control group.

**Table 1:** Baseline characteristics of workers

Characteristic	Number	Mean (SD) or %	
Age	128	46.4 (10.8)	
Gender, male	128	39.8	
Education level	128		
Low		6.3	
Medium		27.3	
High		66.4	
RTW self-efficacy (range 1–6) ^1^	119	3.4 (0.8)	
UCL (Utrecht Coping List)			
Problem-focused coping (range 5–20) ^1^	122	14.2 (2.9)	
Emotional coping (range 5–20) ^1^	123	11.0 (2.7)	
Distraction (range 4–16) ^1^	122	8.5 (2.2)	
4DSQ (Four-Dimensional Symptom Questionnaire)			
Distress (range 0–32) ^1^	121	18.0 (9.4)	
Depression (range 0–12) ^1^	123	2.7 (3.6)	
Anxiety (range 0–24) ^1^	121	5.1 (5.2)	
Somatization (range 0–32) ^1^	119	9.3 (6.4)	
UBOS (Utrecht Burnout Scale–General Survey)			
Burnout exhaustion (range 0–6) ^1^	123	3.9 (1.7)	
Burnout distance (range 0–6) ^1^	123	2.8 (1.7)	
Burnout competence (range 0–6) ^1^	123	3.8 (1.3)	
JCQ (Job Content Questionnaire)			
Psychological job demands (range 12–48) ^1^	120	33.1 (5.7)	
Social support (range 8–32) ^1^	120	22.2 (4.0)	
Decision latitude (range 24–144) ^1^	123	69.9 (8.5)	
Job insecurity (range 3–9) ^1^	119	8.0 (0.8)	

^1^Higher scores indicate a greater presence of the named factor

**Table 2:** RTW-SE means per group per RTW status

		RTW-SE					RTW-SE			
		Intervention group					Control group			
		Baseline		3 months			Baseline		3 months	
		(*n* = 58)		(*n* = 54)			(*n* = 61)		(*n* = 61)	
RTW status	%	Mean	SD	Mean	SD	%	Mean	SD	Mean	SD
Full RTW	32.3	3.65	0.96	4.15	0.63	27.7	3.67	0.51	4.09	0.45
Partial RTW	15.3	3.09	0.68	3.74	0.66	30.8	3.61	0.68	3.87	0.76
No RTW	52.5	3.09	0.61	3.55	0.52	41.5	3.33	1.00	3.50	1.03

*RTW* return-to-work, *RTW-SE* return-to-work self-efficacy

### Does the intervention lead to increased RTW self-efficacy in workers?

An unpaired *t*-test demonstrated that the difference in mean RTW self-efficacy score at baseline between both groups was not significant (t = −1.62, *p* > .05). The model without control for the multilevel effect was found to be the best fitting and was therefore used in the analysis. The results of the linear mixed models analysis are presented in Table 3. In workers in the intervention group, the mean RTW self-efficacy score at baseline was 3.3, which increased by 15 % three months later. In workers who received care as usual, the mean RTW self-efficacy score at baseline was 3.6, which increased by 6 % three months later. The interaction effect between RTW status, intervention and measurements was not significant (*p* ≥ .05) and was therefore removed from the model. There was a significant difference in the RTW self-efficacy score between the group with full RTW and the group with no RTW (t = 3.431, *p* ≤ .05). Controlling for the RTW status, the intervention significantly increased the RTW self-efficacy in workers (t = −2.626, *p* ≤ .05). The Glass’ delta effect size of the increase of RTW self-efficacy by the intervention was 0.51.

**Table 3:** Results of linear mixed models analysis with RTW-SE^a^ and training^b^

	Intervention group				Control group				*P* value^c^
	Baseline		3 months		Baseline		3 months		
	(*n* = 58)		(*n* = 54)		(*n* = 61)		(*n* = 61)		
	Mean	SD	Mean	SD	Mean	SD	Mean	SD	
RTW-SE (range 1–6)	3.30	0.11	3.81	0.10	3.57	0.10	3.78	0.09	0.010*

*RTW-SE* return-to-work self-efficacy

^a^RTW-SE is the dependent variable

^b^Training is the independent variable

^c^ *P* value of the interactive effect of training to the difference between both groups in RTW-SE increases

*significant at *p* ≤ 0.05

### Does the intervention modify the association between RTW self-efficacy and RTW status?

The model without control for the multilevel effect was found to be the best fitting and was therefore used in the analysis. Table 4 shows the results of the generalized linear mixed models analysis, with RTW as the dependent variable, and RTW self-efficacy as the independent variable. A significant association was found between workers’ RTW self-efficacy scores and a full RTW status (t = 2.52, *p* ≤ .05). Compared to workers with lower RTW self-efficacy scores at baseline, workers with higher RTW self-efficacy scores experienced a full RTW three months later significantly more often than they experienced no RTW (OR 2.20, 95 % CI 1.18-4.07). No significant association was found between RTW self-efficacy scores at baseline and a partial RTW three months later. The intervention did not have a modifying effect on the association between RTW self-efficacy baseline and a full RTW. Because the only significant association that was found was between RTW self-efficacy and full RTW, the test for potential confounding factors was performed only for full RTW. None of the tested variables (age, gender, education level, sick leave history, active problem-focused coping, emotional coping, looking for distraction and decreasing tension, distress, depression, anxiety, somatization, burnout, decision latitude, psychological job demands, social support, or job insecurity) caused confounding of the association between RTW self-efficacy baseline and full RTW (data not shown).

**Table 4:** Results of generalized linear mixed models analysis for the associations between RTW-SE^a^ and RTW^b^ (*n* = 116)

	Full RTW			Partial RTW		
Predictor	OR	95 % CI for OR	*P* value	OR	95 % CI for OR	*P* value
RTW-SE	2.20	1.18 – 4.07	0.013*	1.56	0.82 – 2.98	0.174

Reference category: no RTW

*RTW-SE* return-to-work self-efficacy, *RTW* return-to-work

^a^RTW-SE at baseline

^b^RTW three months after the first consultation with the OP

* significant at *p* ≤ 0.05

## Discussion

To our knowledge, this is the first study showing that RTW self-efficacy in sick listed workers can be positively influenced during the first months of sickness absence in a real-life occupational health care setting. The present study showed that the RTW self-efficacy in workers was significantly increased by the intervention, and that RTW status did not influence the increase of RTW self-efficacy. Furthermore, the intervention did not influence the predictive association between the level of RTW self-efficacy at baseline in workers with common mental disorders and RTW status three months after the start of occupational health care. Workers with higher RTW self-efficacy scores at baseline experienced a full RTW significantly more often than did workers with lower RTW self-efficacy baseline scores. Personal factors, mental health factors, or work-related factors at baseline did not influence this association.

The association found between RTW self-efficacy at baseline and actual full RTW three months after the first consultation with an occupational physician is consistent with the findings of previous studies concerning the predictive value of RTW self-efficacy for full RTW [3–5]. In contrast to the findings of Lagerveld and colleagues [4], no association between RTW self-efficacy and a partial RTW was found. However, the mean overall RTW self-efficacy scores at baseline were higher in Lagerveld’s study than they were in the present study (3.8 vs. 3.4). Since the workers in Lagerveld’s study had higher RTW self-efficacy scores at baseline, which is indicative of an earlier RTW, the workers in that study were probably already partially at work three months later, while the workers in our study were still on sick leave at that time. So, this difference in results regarding partial RTW might be explained by the difference in baseline scores. Another explanation for not finding an association between RTW self-efficacy and partial RTW might be a lack of power. Although 22 % of the workers experienced partial RTW, 128 participating workers in our sample might be too little to find an association between RTW self-efficacy and partial RTW.

In contrast with other studies, the present study found that the intervention significantly influenced the increase of RTW self-efficacy over time. Other studies [40, 41] also found a significant increase of RTW self-efficacy over time but this was not caused by the interventions for occupational professionals to guide workers with mental health problems used in these studies. These interventions also concern some kind of problem inventory by the worker making a problem solving plan [40] and/or reintegration plan by the worker [41], homework assignments for the worker [40] and guidance by an occupational physician [40] or an occupational therapist [41]. Unlike the current study one of the other interventions contained group meetings for the workers as well as individual consultations, and roll play experiences for the workers [41]. However, there was one study that also found that the intervention significantly increased RTW self-efficacy, but this intervention was a training for workers with a chronic physical disease [42]. Therefore, this intervention is less comparable to the interventions including a training for occupational health professionals to guide workers with mental health problems in the other studies. Considering these contradictory findings, it seems not easy to influence self-efficacy by the guidance of occupational health care providers. Therefore, more research on innovative interventions is needed to explore the ways in which RTW self-efficacy could be positively influenced.

Based on Bandura’s self-efficacy theory [2], we expected that guidance provided according to the Dutch practice guideline for occupational physicians would contribute to an increase in RTW self-efficacy in workers with common mental disorders. This study showed that the intervention to enhance the guideline-based care provided by occupational physicians significantly increased RTW self-efficacy, as compared to care as usual. The elements in this guideline that contain strategies that are supposed to enhance RTW self-efficacy may indeed have contributed to the increase in RTW self-efficacy in workers with common mental disorders. These findings endorse the potential utility of measuring and seeking to increase RTW self-efficacy in the recovery and RTW process, and could be taken into account by occupational health care providers as they provide guidance to workers with common mental disorders.

Since RTW self-efficacy was only measured at baseline and three months later, we were not able to study the exact course of any changes in RTW self-efficacy within the first three months. It would be worthwhile to evaluate this short-term course of RTW self-efficacy levels and the potential influence exerted by occupational health care, since this can contribute to more knowledge about what happens early in the RTW process and about what might be useful in obtaining an earlier RTW. It would also be worthwhile to evaluate the long-term course of RTW self-efficacy levels and other factors related to an (earlier) RTW, as well as the influence of the training on the development of RTW self-efficacy and other factors related to RTW over time.

A limitation of this study was that RTW self-efficacy was measured after the first consultation with the occupational physician, so workers’ RTW self-efficacy scores could already have been influenced by the guidance of their occupational physicians. Due to the participant enrollment process, workers could only be invited and included in the study after their first consultation with an occupational physician [26]. The first consultation included problem orientation, diagnosis, providing information about the recovery process, and if necessary some initial interventions which could contain elements that were supposed to restore self-efficacy. The first questionnaire was filled out by the worker as soon as possible after his or her first consultation with the occupational physicians. However, measuring RTW self-efficacy some weeks after the start of the sickness absence is comparable to the methods of other studies [4, 5], and was the same for both groups. Nevertheless, RTW self-efficacy at baseline was significantly associated with the occurrence of an actual full RTW, and the intervention significantly influenced increases in RTW self-efficacy over time.

An important limitation of this study was that no objective information was available about the actual guideline adherence of occupational physicians after the training. The occupational physicians were randomly assigned to the intervention group or to the control group through which the influence of other factors on the increase of RTW self-efficacy was not obvious. Although the self-reported guideline adherence by the occupational physicians indicated that their guideline adherence was significantly improved after the training [Joosen et al., submitted, unpublished observations] and self-report measures are highly common in research, objective measures would be preferable and should be used in future research. Therefore, in current study it was not possible to point out which components of the intervention or the provided care contributed to the increases in RTW self-efficacy over time. More research on this important aspect will be needed to learn more about which parts of the guideline and the interventions that influence the recovery and RTW process.

Another limitation of this study was that only those occupational physicians and workers who were willing to participate in the study were included. Probably only occupational physicians who were most eager to improve their guidance of workers with common mental disorders or occupational physicians who were in need of educational credits applied to participate in this study. This might have caused selection bias. Nevertheless, the intervention significantly influenced increases in RTW self-efficacy over time.

This study shows that RTW self-efficacy can be influenced in a real-life Dutch occupational health care setting. Since occupational health care is organized differently in different countries [43], more research is needed to evaluate whether RTW self-efficacy can be influenced in other settings.

## Conclusions

This study contributes to the understanding of the role of RTW self-efficacy in the recovery and RTW process. Since measuring RTW self-efficacy was found to be useful in detecting workers who were at risk for long-term sickness absence in several studies, measured RTW self-efficacy levels can be used to direct the guidance that is offered in the recovery and RTW process. The findings of this study suggest that levels of RTW self-efficacy can be increased during the first months after the start of sick leave with the use of occupational health care, which contains strategies that are supposed to enhance RTW self-efficacy in workers with common mental disorders. This insight contributes to the optimization of the recovery and RTW process and to the development of interventions within occupational health care and guidance.

## Abbreviations

RTW: Return-to-work
ICD-10: International Classification of Diseases version 10
4DSQ: Four dimensional symptom questionnaire
UBOS: Utrecht burnout scale–general survey
UCL: Utrecht coping list
JCQ: Job content questionaire

## References

[1] Bandura A (1977). Self-efficacy: toward a unifying theory of behavioral change. Psychol Rev.

[2] Maddux J, Lewis J, Maddux JE (1995). Self-efficacy and adjustment: basis principles and issues. Self-efficacy, adaptation, and adjustment: theory, research, and application.

[3] Nieuwenhuijsen K, Noordik E, van Dijk FJ, van der Klink JJ (2012). Return to work perceptions and actual return to work in workers with common mental disorders. J Occup Rehabil.

[4] Lagerveld SE, Blonk RWB, Brenninkmeijer V, Schaufeli WB (2010). Return to work among employees with mental health problems: development and validation of a self-efficacy questionnaire. Work Stress.

[5] Brouwer S, Reneman MF, Bultmann U, van der Klink JJ, Groothoff JW (2010). A prospective study of return to work across health conditions: perceived work attitude, self-efficacy and perceived social support. J Occup Rehabil.

[6] Blank L, Peters J, Pickvance S, Wilford J, MacDonald E (2008). A systematic review of the factors which predict return to work for people suffering episodes of poor mental health. J Occup Rehabil.

[7] Hees HL, Koeter MW, Schene AH (2012). Predictors of long-term return to work and symptom remission in sick-listed patients with major depression. J Clin Psychiatry.

[8] Huijs JJ, Koppes LL, Taris TW, Blonk RW (2012). Differences in predictors of return to work among long-term sick-listed employees with different self-reported reasons for sick leave. J Occup Rehabil.

[9] Nielsen MB, Bultmann U, Madsen IE, Martin M, Christensen U, Diderichsen F (2012). Health, work, and personal-related predictors of time to return to work among employees with mental health problems. Disabil Rehabil.

[10] Sampere M, Gimeno D, Serra C, Plana M, Lopez JC, Martinez JM (2012). Return to work expectations of workers on long-term non-work-related sick leave. J Occup Rehabil.

[11] Flach PA, Groothoff JW, Krol B, Bultmann U (2012). Factors associated with first return to work and sick leave durations in workers with common mental disorders. Eur J Public Health.

[12] Cornelius LR, van der Klink JJ, Groothoff JW, Brouwer S (2011). Prognostic factors of long term disability due to mental disorders: a systematic review. J Occup Rehabil.

[13] Lagerveld SE, Bultmann U, Franche RL, van Dijk FJ, Vlasveld MC, van der Feltz-Cornelis CM (2010). Factors associated with work participation and work functioning in depressed workers: a systematic review. J Occup Rehabil.

[14] Nielsen MB, Madsen IE, Bultmann U, Christensen U, Diderichsen F, Rugulies R (2011). Predictors of return to work in employees sick-listed with mental health problems: findings from a longitudinal study. Eur J Public Health.

[15] Brouwers EP, Terluin B, Tiemens BG, Verhaak PF (2009). Predicting return to work in employees sick-listed due to minor mental disorders. J Occup Rehabil.

[16] Brouwer S, Krol B, Reneman MF, Bultmann U, Franche RL, van der Klink JJ (2009). Behavioral determinants as predictors of return to work after long-term sickness absence: an application of the theory of planned behavior. J Occup Rehabil.

[17] van Rhenen W, Schaufeli WB, van Dijk FJ, Blonk RW (2008). Coping and sickness absence. Int Arch Occup Environ Health.

[18] van der Klink JJL, Ausems CMM, Beijderwellen BD, Blonk R, Bruinvels DJ, Dogger J (2007). Richtlijn: Handelen van de berdijfsarts bij werkenden met psychische problemen, herziene versie [guideline: the management of mental health problems of workers by occupational physicians; revised version].

[19] Lugtenberg M, Burgers JS, Westert GP (2009). Effects of evidence-based clinical practice guidelines on quality of care: a systematic review. Qual Saf Health Care.

[20] Grimshaw J, Eccles M, Tetroe J (2004). Implementing clinical guidelines: current evidence and future implications. J Contin Educ Health Prof.

[21] Rebergen DS, Bruinvels DJ, van Tulder MW, van der Beek AJ, van Mechelen W (2009). Cost-effectiveness of guideline-based care for workers with mental health problems. J Occup Environ Med.

[22] Rebergen D, Hoenen J, Heinemans A, Bruinvels D, Bakker A, van Mechelen W (2006). Adherence to mental health guidelines by Dutch occupational physicians. Occup Med.

[23] Vaez M, Rylander G, Nygren Å, Åsberg M, Alexanderson K (2007). Sickness absence and disability pension in a cohort of employees initially on long-term sick leave due to psychiatric disorders in Sweden. Soc Psychiatry Psychiatr Epidemiol.

[24] Dekkers-Sánchez PM, Hoving JL, Sluiter JK, Frings-Dresen MHW (2008). Factors associated with long-term sick leave in sick-listed employees: a systematic review. Occup Environ Med.

[25] Henderson M, Glozier N, Holland EK (2005). Long term sickness absence is caused by common conditions and needs managing. BMJ.

[26] van Beurden KM, Brouwers EP, Joosen MC, Terluin B, van der Klink JJ, van Weeghel J (2013). Effectiveness of guideline-based care by occupational physicians on the return-to-work of workers with common mental disorders: design of a cluster-randomised controlled trial. BMC Public Health.

[27] Korthals AH (2014). Wet- en regelgeving. Wet verbetering poortwachter. [Dutch Gatekeeper Improvement Act].

[28] Grol R, Grimshaw J (2003). From best evidence to best practice: effective implementation of change in patients’ care. Lancet.

[29] Davis D, O’Brien MA, Freemantle N, Wolf FM, Mazmanian P, Taylor-Vaisey A (1999). Impact of formal continuing medical education: do conferences, workshops, rounds, and other traditional continuing education activities change physician behavior or health care outcomes?. JAMA.

[30] Pronovost PJ (2013). Enhancing physicians’ use of clinical guidelines. JAMA.

[31] Cabana MD, Rand CS, Powe NR, Wu AW, Wilson MH, Abboud PA (1999). Why don’t physicians follow clinical practice guidelines? A framework for improvement. JAMA.

[32] Taylor MJ, McNicholas C, Nicolay C, Darzi A, Bell D, Reed JE (2014). Systematic review of the application of the plan-do-study-act method to improve quality in healthcare. BMJ Qual Saf.

[33] Meichenbaum DH, Caneron R, Meichenbaum DH, Jarenko ME (1983). Stress inoculation training. Stress reduction and prevention.

[34] Terluin B, van Marwijk H, Ader H, de Vet H, Penninx B, Hermens M (2006). The Four-Dimensional Symptom Questionnaire (4DSQ): a validation study of a multidimensional self-report questionnaire to assess distress, depression, anxiety and somatization. BMC Psychiatry.

[35] Schaufeli WB, Bakker AB, Hoogduin K, Schaap C, Kladler A (2001). On the clinical validity of the maslach burnout inventory and the burnout measure. Psychol Health.

[36] Schreurs PJG, van de Willige G, Brosschot JF, Tellegen B, Gruas GMH (1993). De Utrechtse Copinglijst: UCL. [The Utrecht Coping List: UCL].

[37] Karasek R, Brisson C, Kawakami N, Houtman I, Bongers P, Amick B (1998). The Job Content Questionnaire (JCQ): an instrument for internationally comparative assessments of psychosocial job characteristics. J Occup Health Psychol.

[38] Verweij A (2013). Scholing en opleiding: indeling opleidingsniveau [education: education level categories]. in: Volksgezondheid Toekomst Verkenning, Nationaal Kompas Volksgezondheid.

[39] Shek DT, Ma CM (2011). Longitudinal data analyses using linear mixed models in SPSS: concepts, procedures and illustrations. Scientific World Journal.

[40] Noordik E, van der Klink JJ, Geskus RB, de Boer MR, van Dijk FJ, Nieuwenhuijsen K (2013). Effectiveness of an exposure-based return-to-work program for workers on sick leave due to common mental disorders: a cluster-randomized controlled trial. Scand J Work Environ Health.

[41] Hees HL, de Vries G, Koeter MW, Schene AH (2013). Adjuvant occupational therapy improves long-term depression recovery and return-to-work in good health in sick-listed employees with major depression: results of a randomised controlled trial. Occup Environ Med.

[42] Varekamp I, Verbeek JH, de Boer A, van Dijk FJ (2011). Effect of job maintenance training program for employees with chronic disease - a randomized controlled trial on self-efficacy, job satisfaction, and fatigue. Scand J Work Environ Health.

[43] World Health Organisation. Global Burden of Disease 2004 update. Geneva: WHO; 2008. http://www.who.int/healthinfo/global_burden_disease/GBD_report_2004update_full.pdf.

